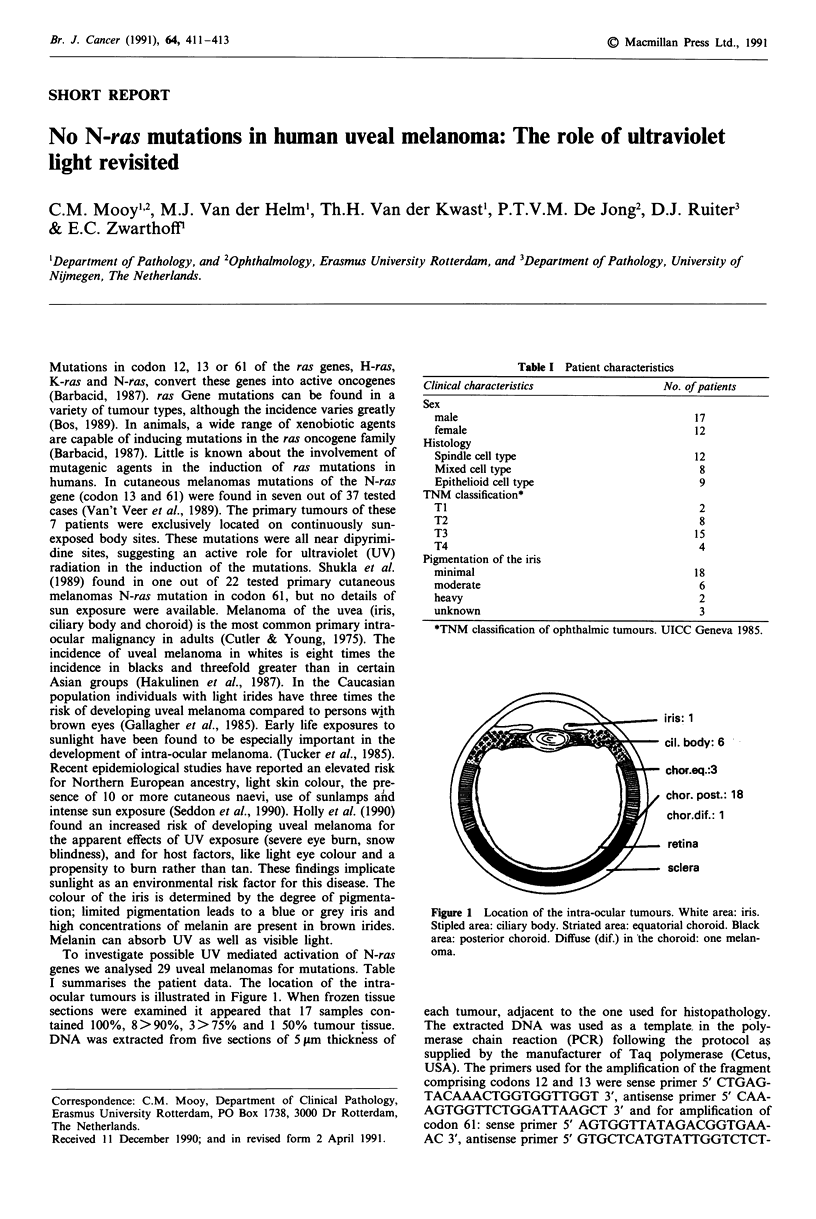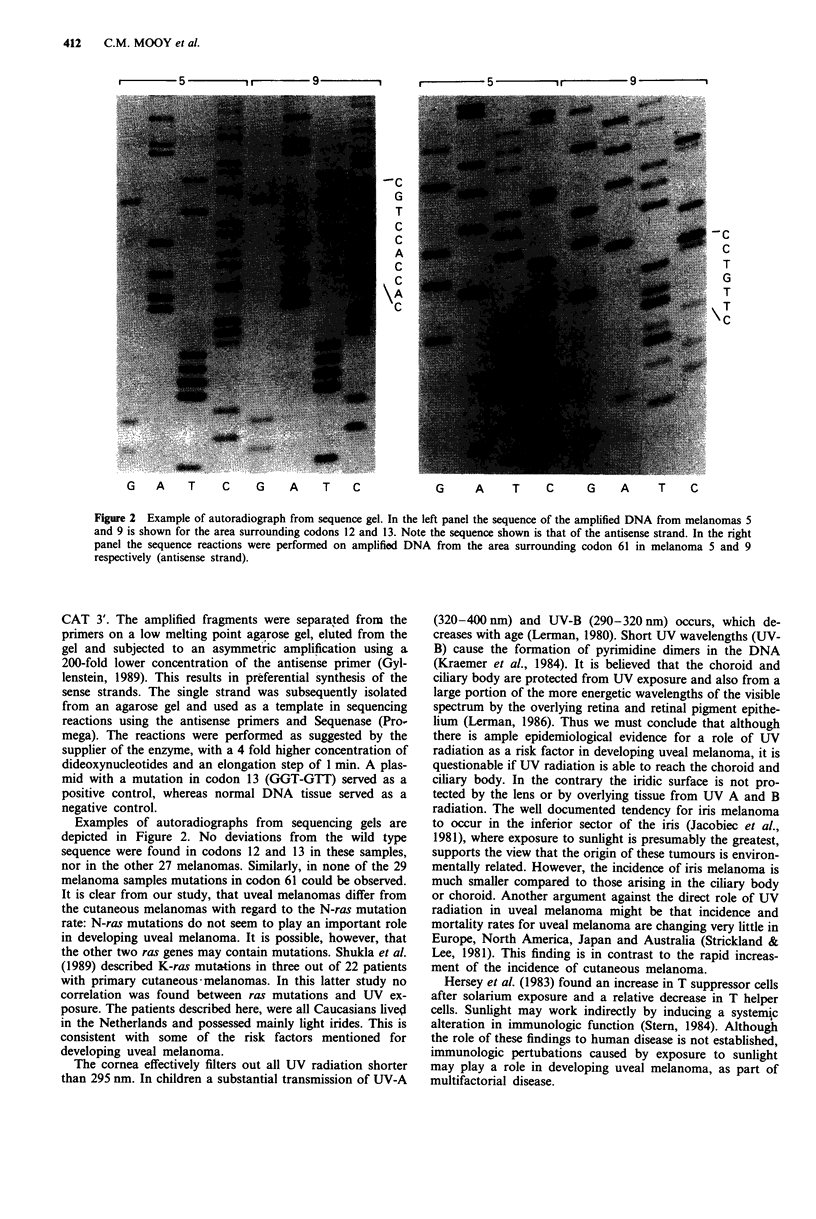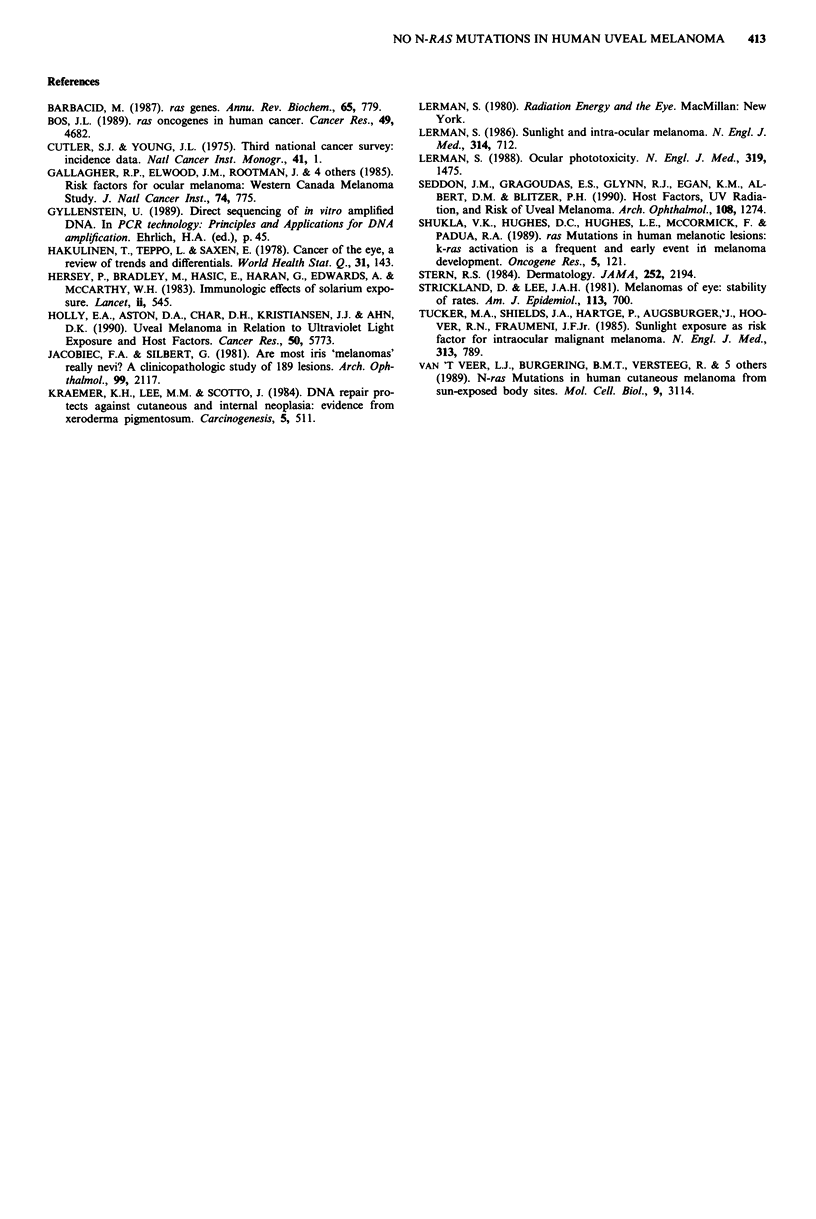# No N-ras mutations in human uveal melanoma: the role of ultraviolet light revisited.

**DOI:** 10.1038/bjc.1991.319

**Published:** 1991-08

**Authors:** C. M. Mooy, M. J. Van der Helm, T. H. Van der Kwast, P. T. De Jong, D. J. Ruiter, E. C. Zwarthoff

**Affiliations:** Department of Pathology, Erasmus University Rotterdam, The Netherlands.

## Abstract

**Images:**


Br. J. Cancer (1991), 64, 411–413 © Macmillan Press Ltd., 1991

SHORT REPORT

### No N-*ras* mutations in human uveal melanoma: The role of ultraviolet light revisited

C.M. Mooy[1,2], M.J. Van der Helm[1], Th.H. Van der Kwast[1], P.T.V.M. De Jong[2], D.J. Ruiter[3] & E.C. Zwarthoff[1]

[1]*Department of Pathology, and* [2]*Ophthalmology, Erasmus University Rotterdam, and* [3]*Department of Pathology, University of Nijmegen, The Netherlands.*

Mutations in codon 12, 13 or 61 of the *ras* genes, H-*ras*, K-*ras* and N-*ras*, convert these genes into active oncogenes (Barbacid, 1987). *ras* Gene mutations can be found in a variety of tumour types, although the incidence varies greatly (Bos, 1989). In animals, a wide range of xenobiotic agents are capable of inducing mutations in the *ras* oncogene family (Barbacid, 1987). Little is known about the involvement of mutagenic agents in the induction of *ras* mutations in humans. In cutaneous melanomas mutations of the N-*ras* gene (codon 13 and 61) were found in seven out of 37 tested cases (Van't Veer *et al.*, 1989). The primary tumours of these 7 patients were exclusively located on continuously sun-exposed body sites. These mutations were all near dipyrimidine sites, suggesting an active role for ultraviolet (UV) radiation in the induction of the mutations. Shukla *et al.* (1989) found in one out of 22 tested primary cutaneous melanomas N-*ras* mutation in codon 61, but no details of sun exposure were available. Melanoma of the uvea (iris, ciliary body and choroid) is the most common primary intra-ocular malignancy in adults (Cutler & Young, 1975). The incidence of uveal melanoma in whites is eight times the incidence in blacks and threefold greater than in certain Asian groups (Hakulinen *et al.*, 1987). In the Caucasian population individuals with light irides have three times the risk of developing uveal melanoma compared to persons with brown eyes (Gallagher *et al.*, 1985). Early life exposures to sunlight have been found to be especially important in the development of intra-ocular melanoma. (Tucker *et al.*, 1985). Recent epidemiological studies have reported an elevated risk for Northern European ancestry, light skin colour, the presence of 10 or more cutaneous naevi, use of sunlamps and intense sun exposure (Seddon *et al.*, 1990). Holly *et al.* (1990) found an increased risk of developing uveal melanoma for the apparent effects of UV exposure (severe eye burn, snow blindness), and for host factors, like light eye colour and a propensity to burn rather than tan. These findings implicate sunlight as an environmental risk factor for this disease. The colour of the iris is determined by the degree of pigmentation; limited pigmentation leads to a blue or grey iris and high concentrations of melanin are present in brown irides. Melanin can absorb UV as well as visible light.

To investigate possible UV mediated activation of N-*ras* genes we analysed 29 uveal melanomas for mutations. Table I summarises the patient data. The location of the intra-ocular tumours is illustrated in Figure 1. When frozen tissue sections were examined it appeared that 17 samples contained 100%, 8>90%, 3>75% and 1 50% tumour tissue. DNA was extracted from five sections of 5 μm thickness of each tumour, adjacent to the one used for histopathology. The extracted DNA was used as a template in the polymerase chain reaction (PCR) following the protocol as supplied by the manufacturer of Taq polymerase (Cetus, USA). The primers used for the amplification of the fragment comprising codons 12 and 13 were sense primer 5′ CTGAG-TACAAACTGGTGGTTGGT 3′, antisense primer 5′ CAA-AGTGGTTCTGGATTAAGCT 3′ and for amplification of codon 61: sense primer 5′ AGTGGTTATAGACGGTGAA-AC 3′, antisense primer 5′ GTGCTCATGTATTGGTCTCT-

**Table I** Patient characteristics

| *Clinical characteristics* | *No. of patients* |
|---|---|
| Sex | |
| male | 17 |
| female | 12 |
| Histology | |
| Spindle cell type | 12 |
| Mixed cell type | 8 |
| Epithelioid cell type | 9 |
| TNM classification* | |
| T1 | 2 |
| T2 | 8 |
| T3 | 15 |
| T4 | 4 |
| Pigmentation of the iris | |
| minimal | 18 |
| moderate | 6 |
| heavy | 2 |
| unknown | 3 |

*TNM classification of ophthalmic tumours. UICC Geneva 1985.

**Figure 1** Location of the intra-ocular tumours. White area: iris. Stipled area: ciliary body. Striated area: equatorial choroid. Black area: posterior choroid. Diffuse (dif.) in the choroid: one melanoma.

Correspondence: C.M. Mooy, Department of Clinical Pathology, Erasmus University Rotterdam, PO Box 1738, 3000 Dr Rotterdam, The Netherlands.
Received 11 December 1990; and in revised form 2 April 1991.

**Figure 2** Example of autoradiograph from sequence gel. In the left panel the sequence of the amplified DNA from melanomas 5 and 9 is shown for the area surrounding codons 12 and 13. Note the sequence shown is that of the antisense strand. In the right panel the sequence reactions were performed on amplified DNA from the area surrounding codon 61 in melanoma 5 and 9 respectively (antisense strand).

CAT 3'. The amplified fragments were separated from the primers on a low melting point agarose gel, eluted from the gel and subjected to an asymmetric amplification using a 200-fold lower concentration of the antisense primer (Gyllenstein, 1989). This results in preferential synthesis of the sense strands. The single strand was subsequently isolated from an agarose gel and used as a template in sequencing reactions using the antisense primers and Sequenase (Promega). The reactions were performed as suggested by the supplier of the enzyme, with a 4 fold higher concentration of dideoxynucleotides and an elongation step of 1 min. A plasmid with a mutation in codon 13 (GGT-GTT) served as a positive control, whereas normal DNA tissue served as a negative control.

Examples of autoradiographs from sequencing gels are depicted in Figure 2. No deviations from the wild type sequence were found in codons 12 and 13 in these samples, nor in the other 27 melanomas. Similarly, in none of the 29 melanoma samples mutations in codon 61 could be observed. It is clear from our study, that uveal melanomas differ from the cutaneous melanomas with regard to the N-*ras* mutation rate: N-*ras* mutations do not seem to play an important role in developing uveal melanoma. It is possible, however, that the other two *ras* genes may contain mutations. Shukla *et al.* (1989) described K-*ras* mutations in three out of 22 patients with primary cutaneous melanomas. In this latter study no correlation was found between *ras* mutations and UV exposure. The patients described here, were all Caucasians lived in the Netherlands and possessed mainly light irides. This is consistent with some of the risk factors mentioned for developing uveal melanoma.

The cornea effectively filters out all UV radiation shorter than 295 nm. In children a substantial transmission of UV-A (320–400 nm) and UV-B (290–320 nm) occurs, which decreases with age (Lerman, 1980). Short UV wavelengths (UV-B) cause the formation of pyrimidine dimers in the DNA (Kraemer *et al.*, 1984). It is believed that the choroid and ciliary body are protected from UV exposure and also from a large portion of the more energetic wavelengths of the visible spectrum by the overlying retina and retinal pigment epithelium (Lerman, 1986). Thus we must conclude that although there is ample epidemiological evidence for a role of UV radiation as a risk factor in developing uveal melanoma, it is questionable if UV radiation is able to reach the choroid and ciliary body. In the contrary the iridic surface is not protected by the lens or by overlying tissue from UV A and B radiation. The well documented tendency for iris melanoma to occur in the inferior sector of the iris (Jacobiec *et al.*, 1981), where exposure to sunlight is presumably the greatest, supports the view that the origin of these tumours is environmentally related. However, the incidence of iris melanoma is much smaller compared to those arising in the ciliary body or choroid. Another argument against the direct role of UV radiation in uveal melanoma might be that incidence and mortality rates for uveal melanoma are changing very little in Europe, North America, Japan and Australia (Strickland & Lee, 1981). This finding is in contrast to the rapid increasment of the incidence of cutaneous melanoma.

Hersey *et al.* (1983) found an increase in T suppressor cells after solarium exposure and a relative decrease in T helper cells. Sunlight may work indirectly by inducing a systemic alteration in immunologic function (Stern, 1984). Although the role of these findings to human disease is not established, immunologic pertubations caused by exposure to sunlight may play a role in developing uveal melanoma, as part of multifactorial disease.

### References

BARBACID, M. (1987). *ras* genes. *Annu. Rev. Biochem.*, **65,** 779.

BOS, J.L. (1989). *ras* oncogenes in human cancer. *Cancer Res.*, **49,** 4682.

CUTLER, S.J. & YOUNG, J.L. (1975). Third national cancer survey: incidence data. *Natl Cancer Inst. Monogr.*, **41,** 1.

GALLAGHER, R.P., ELWOOD, J.M., ROOTMAN, J. & 4 others (1985). Risk factors for ocular melanoma: Western Canada Melanoma Study. *J. Natl Cancer Inst.*, **74,** 775.

GYLLENSTEIN, U. (1989). Direct sequencing of *in vitro* amplified DNA. In *PCR technology: Principles and Applications for DNA amplification*. Ehrlich, H.A. (ed.), p. 45.

HAKULINEN, T., TEPPO, L. & SAXEN, E. (1978). Cancer of the eye, a review of trends and differentials. *World Health Stat. Q.*, **31,** 143.

HERSEY, P., BRADLEY, M., HASIC, E., HARAN, G., EDWARDS, A. & McCARTHY, W.H. (1983). Immunologic effects of solarium exposure. *Lancet*, **ii,** 545.

HOLLY, E.A., ASTON, D.A., CHAR, D.H., KRISTIANSEN, J.J. & AHN, D.K. (1990). Uveal Melanoma in Relation to Ultraviolet Light Exposure and Host Factors. *Cancer Res.*, **50,** 5773.

JACOBIEC, F.A. & SILBERT, G. (1981). Are most iris 'melanomas' really nevi? A clinicopathologic study of 189 lesions. *Arch. Ophthalmol.*, **99,** 2117.

KRAEMER, K.H., LEE, M.M. & SCOTTO, J. (1984). DNA repair protects against cutaneous and internal neoplasia: evidence from xeroderma pigmentosum. *Carcinogenesis*, **5,** 511.

LERMAN, S. (1980). *Radiation Energy and the Eye*. MacMillan: New York.

LERMAN, S. (1986). Sunlight and intra-ocular melanoma. *N. Engl. J. Med.*, **314,** 712.

LERMAN, S. (1988). Ocular phototoxicity. *N. Engl. J. Med.*, **319,** 1475.

SEDDON, J.M., GRAGOUDAS, E.S., GLYNN, R.J., EGAN, K.M., ALBERT, D.M. & BLITZER, P.H. (1990). Host Factors, UV Radiation, and Risk of Uveal Melanoma. *Arch. Ophthalmol.*, **108,** 1274.

SHUKLA, V.K., HUGHES, D.C., HUGHES, L.E., McCORMICK, F. & PADUA, R.A. (1989). *ras* Mutations in human melanotic lesions: k-*ras* activation is a frequent and early event in melanoma development. *Oncogene Res.*, **5,** 121.

STERN, R.S. (1984). Dermatology. *JAMA*, **252,** 2194.

STRICKLAND, D. & LEE, J.A.H. (1981). Melanomas of eye: stability of rates. *Am. J. Epidemiol.*, **113,** 700.

TUCKER, M.A., SHIELDS, J.A., HARTGE, P., AUGSBURGER, J., HOOVER, R.N., FRAUMENI, J.F.Jr. (1985). Sunlight exposure as risk factor for intraocular malignant melanoma. *N. Engl. J. Med.*, **313,** 789.

VAN 'T VEER, L.J., BURGERING, B.M.T., VERSTEEG, R. & 5 others (1989). N-*ras* Mutations in human cutaneous melanoma from sun-exposed body sites. *Mol. Cell. Biol.*, **9,** 3114.